# Using Geographical Information Systems to Identify Populations in Need of Improved Accessibility to Antivenom Treatment for Snakebite Envenoming in Costa Rica

**DOI:** 10.1371/journal.pntd.0002009

**Published:** 2013-01-31

**Authors:** Erik Hansson, Mahmood Sasa, Kristoffer Mattisson, Arodys Robles, José María Gutiérrez

**Affiliations:** 1 Occupational and Environmental Medicine, Lund University, Lund, Sweden; 2 Instituto Clodomiro Picado, Facultad de Microbiogía, Universidad de Costa Rica, San José, Costa Rica; 3 Centro Centroamericano de Población, Universidad de Costa Rica, San José, Costa Rica; Institut de Recherche pour le Développement, Benin

## Abstract

**Introduction:**

Snakebite accidents are an important health problem in rural areas of tropical countries worldwide, including Costa Rica, where most bites are caused by the pit-viper *Bothrops asper*. The treatment of these potentially fatal accidents is based on the timely administration of specific antivenom. In many regions of the world, insufficient health care systems and lack of antivenom in remote and poor areas where snakebites are common, means that efficient treatment is unavailable for many snakebite victims, leading to unnecessary mortality and morbidity. In this study, geographical information systems (GIS) were used to identify populations in Costa Rica with a need of improved access to antivenom treatment: those living in areas with a high risk of snakebites and long time to reach antivenom treatment.

**Method/Principal Findings:**

Populations living in areas with high risk of snakebites were identified using two approaches: one based on the district-level reported incidence, and another based on mapping environmental factors favoring *B. asper* presence. Time to reach treatment using ambulance was estimated using cost surface analysis, thereby enabling adjustment of transportation speed by road availability and quality, topography and land use. By mapping populations in high risk of snakebites and the estimated time to treatment, populations with need of improved treatment access were identified.

**Conclusion/Significance:**

This study demonstrates the usefulness of GIS for improving treatment of snakebites. By mapping reported incidence, risk factors, location of existing treatment resources, and the time estimated to reach these for at-risk populations, rational allocation of treatment resources is facilitated.

## Author Summary

Snakebite accidents are a neglected cause of much death and suffering worldwide. The situation is especially severe in rural areas of low income tropical countries, where long distances to well-equipped health care facilities mean that the time needed to reach antivenom treatment is often long. Delays in receiving antivenom treatment of snakebites could lead to severe outcomes, such as death or permanent disability. In this study we demonstrate how Geographical Information Systems (GIS) could be used to allocate antivenom rationally and thereby decrease the impact of snakebite in a cost-effective manner. We map areas with a high risk of snakebite accidents, based on a high reported incidence and environmental conditions favoring snakebites. We then estimate the time needed to reach a facility in which antivenom treatment is available for the population in these high risk areas, thus identifying areas in need of improved treatment accessibility. Based on these maps of the unmet need of antivenom treatment, allocation of antivenom and other resources needed to treat snakebites can be made more efficiently.

## Introduction

Snakebites are a health problem in several parts of the world [1], especially affecting poor people living in rural areas in tropical countries [2]. The treatment of snakebite envenoming is based on the timely administration of animal-derived antivenoms [3]. However, a number of factors limit the accessibility of antivenoms in various areas of the world [3], [4]. The relatively high cost of some antivenoms make accessing them difficult in low income countries [4]. In addition, long distances to healthcare facilities and incorrect distribution of antivenoms within countries [3], [5], [6] means that for many of the snakebite victims worldwide, specific treatment with safe and effective antivenoms is unavailable, and traditional healers are instead often consulted [1], [7]–[9]. This is an unfortunate situation as antivenom treatment is highly effective at preventing morbidity and mortality caused by snakebite envenoming [3]. In other regions of the world, such as in various countries in Latin America, antivenom is readily available in health centers [5]. This is the case of Costa Rica, where antivenom is widely available throughout the public health system, as a result of domestic production and effective acquisition and distribution schemes [5].

The incidence of snakebites is known to vary widely on a sub-national level due to environmental and demographic factors [10]–[14]. In Central America, an estimated number of 4,000 snakebite cases occur every year [15]. The Central America lancehead pitviper *Bothrops asper*, locally known as ‘terciopelo’ or ‘barba amarilla’, which is widely distributed in humid, lowland areas (0–600 m.a.s.l., but it might be found up to 1,200 m.a.s.l.) [16], [17], is responsible for 50–80% of the bites and 60–90% of all fatal cases in Central America and northern South America [13]. Epidemiological studies indicate that most victims are agricultural workers and/or rural residents in general [13]. In the 1990s, the snakebite incidence in Costa Rica was approximately 15 per 100,000 inhabitants per year, with a decreasing trend [14]. Snakebite mortality has also been reported to decrease in Costa Rica, an observation attributed to, among other things, improvements in the health care system, including better geographical accessibility to health care facilities [12], [18]. The use of first aid interventions and the attendance to traditional healers for treatment of snakebites is very limited in Costa Rica, at least among those seeking hospital treatment [19].

Identification of areas with high snakebite incidence is an important goal for the design of preventive and therapeutic interventions aimed at reducing the impact of snakebites. Such interventions could include educational programs, antivenom distribution and training of health staff in the management of these envenomations [4]–[6]. Similar issues of rational distribution of treatment and intervention resources are being considered in the struggle against other neglected tropical diseases, with geographical information systems (GIS) increasingly being used as a tool for improved decision-making [20]. Leynaud and Reati [21] described the use of the free GIS-software SIGEpi [22] for identification of areas with a high risk of snakebites and long distances to healthcare in Argentina. It is necessary to further extend the use of this methodology in other countries, in order to get an integrated view of snakebite envenomings on various regions of the world.

The health care system in Costa Rica is considered well developed, in terms of geographical accessibility [23] and insurance coverage [24]. This country has made important achievements in public health, such as long life expectancy and low infant mortality rate [24], [25]. Antivenom is available in all clinics and hospitals and, according to a recent decision by the state social insurance administration that runs all public health services in the country (Caja Costarricense del Seguro Social, henceforth CCSS), it can also be distributed to primary health care teams (Equipos Básicos de Atención Integral en Salud, EBAIS) of which there are approximately 1 per 5,000 inhabitants [24]. This opens the possibility to further improve the accessibility to antivenoms in Costa Rica. However, the distribution of antivenoms to EBAIS has to be carefully analyzed, in order to ensure that mostly EBAIS serving a population with high snakebite risk and limited access to clinics and hospitals will receive antivenoms. This will prevent unnecessary wastage of this precious drug by deploying it to regions where snakebites are infrequent, or where distances to hospitals or clinics where these accidents are treated are short.

### Objectives

The primary aim of the present study is to provide information to assist decision-making concerning for which primary health care facilities (EBAIS) it is suitable to have antivenom, i.e. those that serve a population with a high risk of snakebites and long transport times to hospitals or clinics where antivenom is available. The methodology used in this study might be applied in other countries and regions where snakebite envenoming is a relevant public health hazard, in order to identify vulnerable areas that require interventions aimed at ensuring the access to antivenom and adequate medical treatment. As part of the primary aim, we will demonstrate the benefits of spatial smoothing of the small-area snakebite incidence data for increasing the interpretability of this data. Aside from the primary aim, we will also describe the relationship between some district-level environmental and demographic factors and snakebite incidence in Costa Rica.

## Materials and Methods

### Outline of the analysis

Populations in a high risk of snakebite were identified using two approaches: one based on the reported district-level snakebite incidence, and another based on identifying populations with environmental and demographic risk factors favoring snakebites.

For the first approach, an incidence of 30 bites per 100,000 population per year was selected as threshold, on the basis of the overall incidence of snakebite envenomings in Costa Rica (15 cases per 100,000 inhabitants per year [14]). In order to reduce the random noise in the small-area incidence, a Bayesian smoothing method [26] was employed by fitting a Poisson regression to the observed incidence in the period 2003–2007. Smoothing can be explained as modelling the underlying risk around which the observed incidence varies stochastically. As such the smoothed incidence estimates give a more stable picture of the actual risk of snakebites than the raw incidence data, making interpretation easier and giving a better basis for decision-making [26]. Spatial smoothing was also used by Leynaud and Reati [21] in their study of snakebite epidemiology in Argentina. In this study, we present an alternative smoothing method that includes also measured explanatory variables, and allows for estimation of the probability that an incidence threshold is exceeded [27]. We then compare the performance of this smoothing method with the one used by Leynaud and Reati [21]. Measured variables to be included in the smoothing were identified in the literature as important factors influencing snake presence, i.e. environmental factors [16], and snake-human interaction, in this case urban population percentage [13].

For the second approach to identifying areas in possible need of improved antivenom accessibility, rural areas in humid, lowland areas were considered at high risk of snakebites, as this is a habitat suitable for the medically most relevant snake species *B. asper* [16].

Among the populations identified as in high risk of snakebites by either of these two approaches, those with an estimated minimum transportation time to hospital or clinic exceeding 2 hours were identified as those requiring improved antivenom accessibility, based on the time identified as critical to avoid mortality from snakebites [13].

### Material

The centroid of the census enumeration units were used to estimate the location of the population [23]. A 2 km buffer around the census tract centroids were used as a proxy for populated areas. Census enumeration unit population size in year 2000 and information whether the unit was urban or rural was available as attribute information.

**Figure 1:**
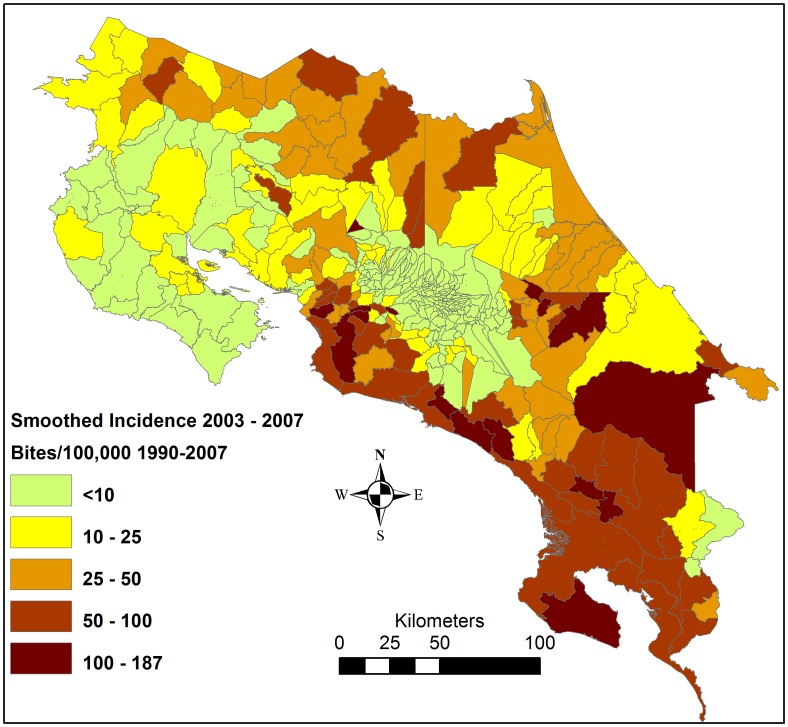
Snakebite incidence per 100,000 inhabitants per district 1990–2007.

Information about snakebite cases 1990–2007 per district of residence were obtained from hospital discharge reports from the CCSS. Duplicate cases, with exactly the same age, sex, month of bite and district of residence (n = 123) were removed from the analysis. Cases reported from health care facilities in which it was highly unlikely that the place of the bite was in the district of residence (n = 141), or when the place of residence was unknown (n = 43) were also removed. These steps left 9,149 snakebites, divided over 413 districts (the smallest administrative area) (Figure 1). The location of *B. asper* specimens (n = 241) collected to the serpentarium at Instituto Clodomiro Picado, San José, Costa Rica was obtained.

Digital maps of environmental variables (elevation 30×30 m raster, mean annual precipitation, number of dry months, forest coverage, and biotic unit [28]) were obtained from a database compiled by Instituto Tecnológico de Costa Rica [29]. The values for each of the environmental variables were extracted to the census enumeration unit centroids, and the population-weighted district average value for each of the environmental variables was calculated. For forest coverage, the proportion of inhabitants living within 500 meters from forests larger than 5 ha was calculated. A few districts (n = 9) were covered by clouds in the satellite image from which the forest coverage was derived, and these were assigned the mean forest coverage value of the other districts.

The location of lakes, rivers, clinics, hospitals and roads were obtained from the same database as the environmental variables [29]. A list of primary health care facilities, i.e. Equipos Básicos de Atención Integral en Salud (EBAIS) was obtained from the CCSS [30]. EBAIS were geocoded by matching the name and service area of the facility with the name and district of communities in the country, available as a digital map [29]. In most cases, there was a community with the same name as the facility. If there was no such community, the facility was located in the main community of the district. If it was not possible to determine which the main community was, the facility was located in one of the communities located in the center of the service area. Red Cross ambulance stations were geo-coded using a list and map available at the Costa Rican Red Cross Website [31].

### A Bayesian Poisson regression model of snakebite incidence

In order to analyze which factors were important for snakebite occurrence, and to smooth the snakebite incidence, a Bayesian Poisson regression was used to model the district-level risk of snakebites. Factors chosen for inclusion were those that had previously been identified in literature as either influencing snake presence [16] or important for snake-human interaction patterns [13]. The number of snakebite cases *Y* per district *i* were modeled as Poisson variates in the form;
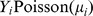


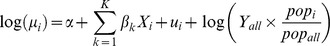
where 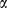 is the intercept, 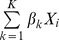 a matrix of five fixed effect district-level explanatory variables (urbanity, forest coverage, elevation, precipitation, and number of dry months), 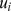 a spatially correlated random effect modelled using a conditional autoregressive (CAR) prior structure [32], which assumed dependence between districts if they shared a border or corner, and 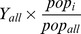 correspond to the number of snakebites that would be expected if they were distributed evenly within the population, i.e. the offset. In order to facilitate convergence, continuous variable (precipitation, number of dry months and elevation) were standardized to have mean 0 and standard deviation 1. The coefficients for the fixed effects were assigned non-informative normal distribution priors (mean 0 and precision 0.0001), and the intercept a non-informative flat prior (range −∞ to ∞). The variance of the spatially correlated random effects was assigned a non-informative gamma prior. The model was fitted in WinBUGS 1.4.3 [33].

When the district-level risk factors of snakebites were analyzed, all snakebite data (i.e. 1990–2007) were used. Convergence was reached after 50 000 iterations, after which another 100 000 iterations were performed to estimate the posterior distribution, from which model parameters with 95% credible intervals (Cr.I.) were obtained.

### Demonstration of spatial smoothing of small-area snakebite incidence data

The snakebite incidence dataset was divided into nine temporal periods, six training periods on which the smoothing was performed, and three test periods used to assess the ability of the estimates produced by the smoothing methods to improve identification of future high-incidence districts. Three of the training periods were five-year periods (1990–1994, 1994–1998 and 1998–2002) and three one-year periods (1994, 1998 and 2002).The three test periods were five years (1995–1999, 1999–2003 and 2003–2007).

The first smoothing method (A) was the same Bayesian model with random spatial effects and fixed effect explanatory variables (elevation, number of dry months, precipitation, forest coverage and urban population percentage) as described above. The Bayesian framework makes it possible to estimate the probability that the incidence exceeds a threshold directly from the posterior distribution. For each of the training time periods, the models were run until convergence was reached (after 50,000 iterations) and then for another 50,000 iterations to obtain samples from the posterior distribution. From the posterior distribution, the probability that the incidence exceeded 30 bites per 100,000 inhabitants was calculated.

The second smoothing method (B) was the one used by Leynaud and Reati [21]; the tool for automated spatial Bayesian smoothing of incidence rates (“Suavizador espacial de tasas”) available in SIGEpi [22]. The settings of local smoothing and neighborhood defined as adjacency were used.

The abilities of these smoothed estimates, and the unsmoothed incidence, to identify whether the incidence in a district would exceed 30 bites per 100,000 in the next-coming five-year period (i.e. the test periods 1995–1999, 1999–2003 and 2003–2007) were assessed using the ROC Curve function of IBM SPSS 20 [34]. The AUC (area under the curve) of the ROC (receiver operating characteristics; a plot of sensitivity vs. one minus specificity) is an often used tool to assess the discriminatory ability of tests; an AUC of 0.7–0.9 indicate reasonable discriminatory ability, and >0.9 very good discriminatory ability [35]. For each of the six combinations of training time (1 and 5 years) and smoothing method (A, B and unsmoothed), the mean AUC (with 95% empirical confidence intervals (C.I.)) were computed by simulating plausible AUC values from the uncertainty interval for the three training-test period pairs and calculating the mean of these.

### Identification of high-risk areas

**Figure 2:**
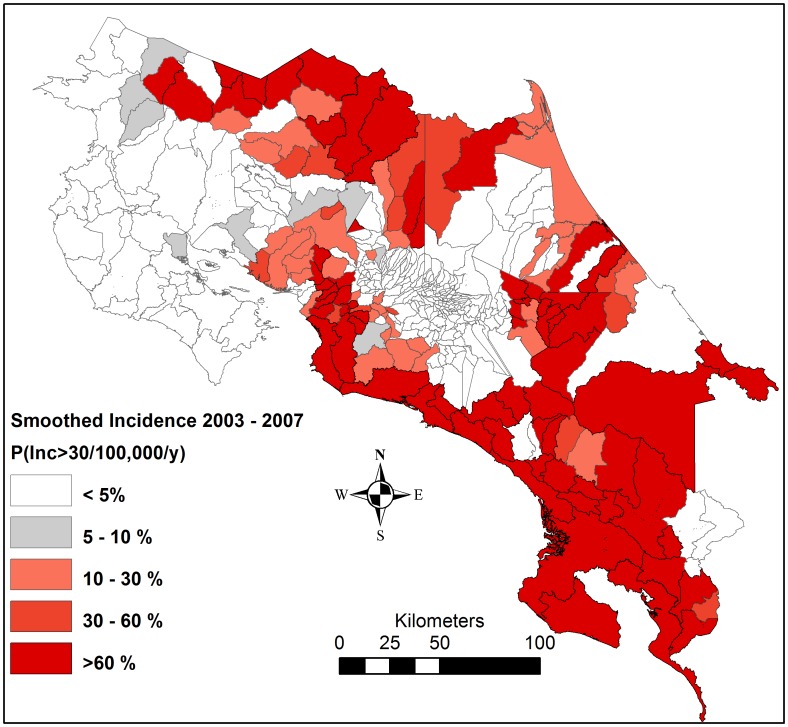
The probability that the smoothed incidence exceeded 30 bites per 100,000 inhabitants in 2003–2007. Inc = incidence.

In order to produce smoothed estimates of the underlying snakebite risk in a district, that would be less affected by random noise, and thereby able to more precisely identify the need for antivenom accessibility in that district in the upcoming years, the above model was applied to the most recently available 5-year-period of the snakebite data; i.e. 2003–2007. After reaching convergence after 50,000 iterations, the model was run for another 50,000 iterations, during which the probability that the smoothed incidence exceeded the threshold of 30 bites per 100,000 inhabitants was calculated (Figure 2).

**Figure 3:**
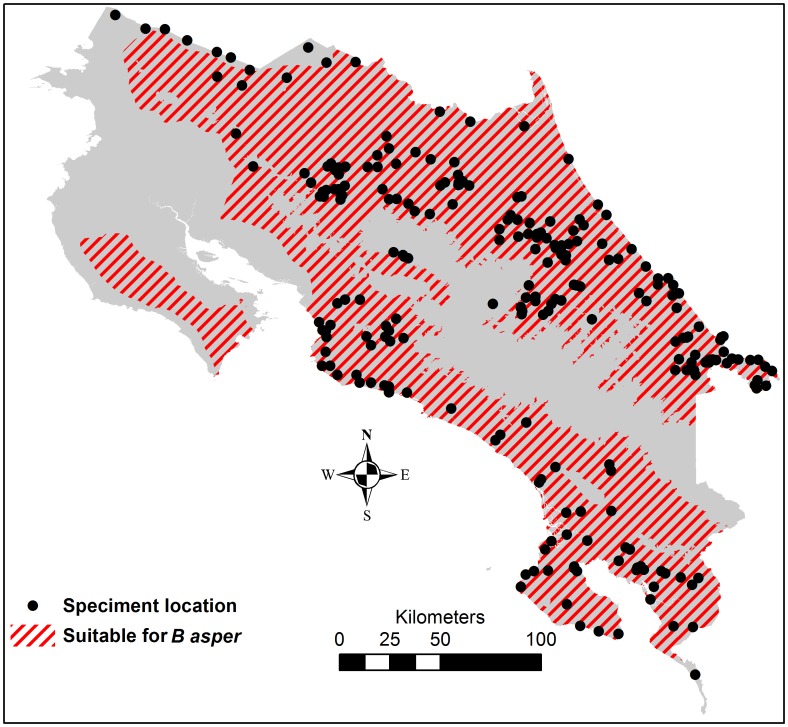
Geographical extent of habitat suitable for *Bothrops asper* and location of specimens collected at ICP. ICP = Instituto Clodomiro Picado.

**Figure 4:**
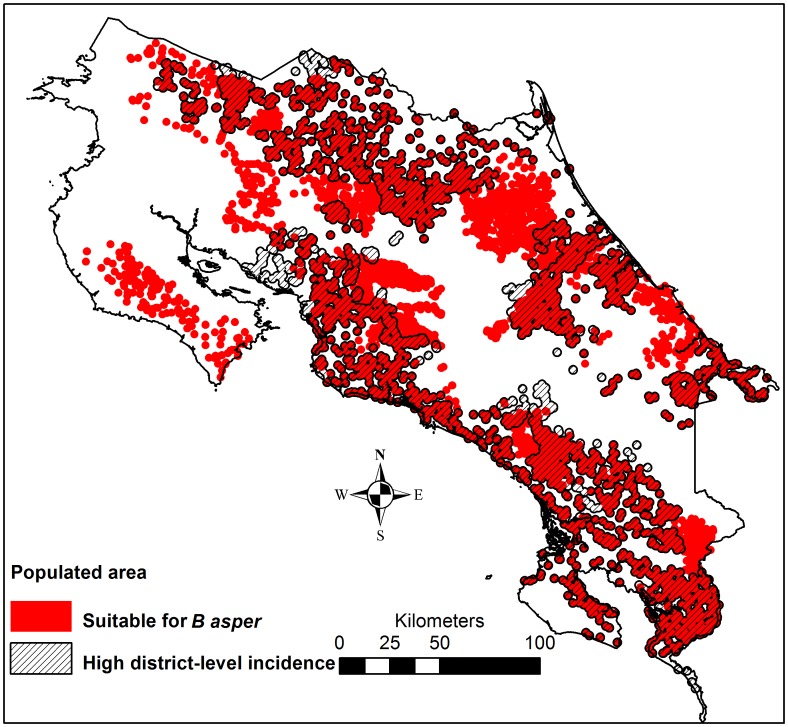
Populated areas in high risk of snakebites. Populated areas = areas within 2 km from a census tract centroid in a high risk of snakebites, i.e. in districts with a high snakebite incidence, and/or in an area suitable for *B. asper*.

The incidence threshold exceedance probability estimate that corresponded to 90% sensitivity in detecting districts with an incidence above the 30 bites per 100,000 inhabitants threshold in a future 5-year period was on average 10% for three earlier 5-year periods (data not shown). Therefore, this was set as the cut-off probability for identifying high-risk districts, in need of good antivenom accessibility. Rural population residing in an environment suitable for the medically most relevant snake species *B. asper* (i.e. below 1200 meters of elevation and in Moist, Wet or Pluvial biotic unit [16], [28], [29]) (Figure 3) were also identified as living in high-risk areas (Figure 4).

### Identification of high-risk areas in need of improved treatment accessibility

The mountainous terrain of Costa Rica imposes strong restrictions on human movement. The Euclidean distance (straight line) approximation of the time needed to move from the place of snakebite to the healthcare facility (hospital or clinic) might therefore not accurately capture the real time spent in this transportation. We aimed at constructing a model of the time needed to reach antivenom treatment, i.e. hospitals or clinics, which takes into account the availability and quality of roads, topography, land cover and the mode of transportation used to reach healthcare. We assumed that people would choose ambulance services after a snakebite accident. Travel time was therefore calculated as time with ambulance from closest ambulance station, to place of residence, and from there to closest hospital or clinic. The time estimated should be considered a minimum, ideal, time as it is modeled assuming that the ambulance leaves immediately after the snakebite to meet up the snakebite victim, without any delays.

The time to treatment was estimated using GRASS 6.4.1 [36]. The road data vector layer was converted to a 30×30 m raster and classified according to the following assumed speeds: primary roads, 60 km/h; secondary and urban roads, 40 km/h; and tertiary, local and other roads, 20 km/h. Off-road speed was set to 6 km/h, but the off-road speed will become 3 km/h as this distance is not covered by ambulance transportation and it will be counted twice (see below). Off-road speed in areas covered by forest was assumed to be 50% slower. Streams, rivers and lakes, that were not crossed by roads, were given a speed one fifth of that in open terrain, in order to penalize movement across water. From the elevation raster, a slope raster was constructed. The road/off-road speed raster, and the slope raster were combined to yield a raster of the time needed to travel one raster cell (*t_r,α_*) using Equation 1.



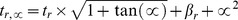
(Equation 1)The first part (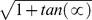) of Equation 1 calculates the excess distance needed to travel the cell due to change in altitude (i.e. the hypotenuse). The second part (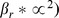
of the equation aims at taking into account the reduced speed associated with moving in undulated terrain, as well as to create barriers in extremely steep slopes. In this part of the equation, we assume that the excess time needed to travel one of the 30 m cells is proportional to the squared slope 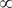 (in degrees) multiplied by a coefficient *β_r_* (0.001 for road travel and 0.02 for off-road). The value of this co-efficient was chosen based on simulation of what values produced reasonable estimations.

**Figure 5:**
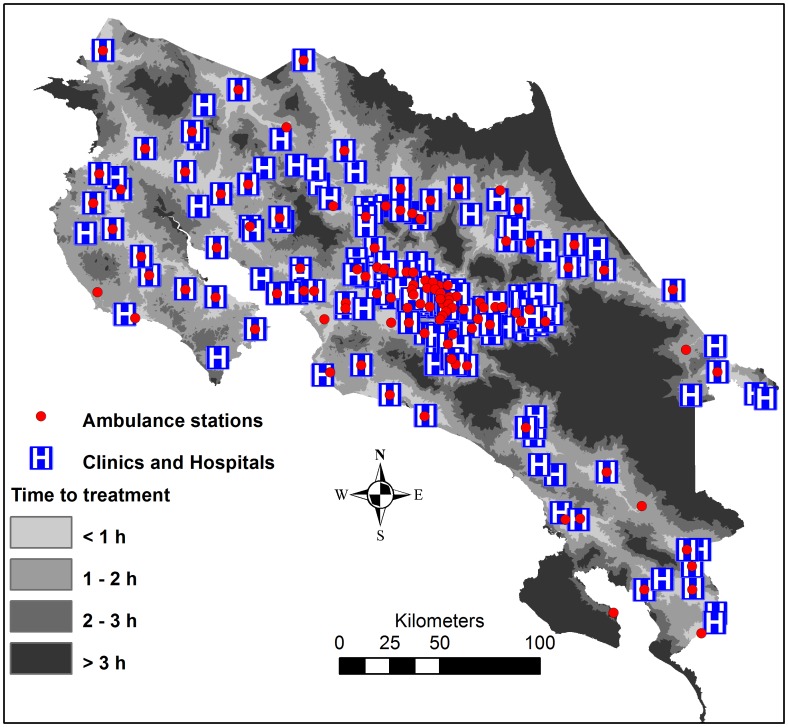
Clinics, hospitals, ambulance stations and estimated time to hospital or clinic.

**Figure 6:**
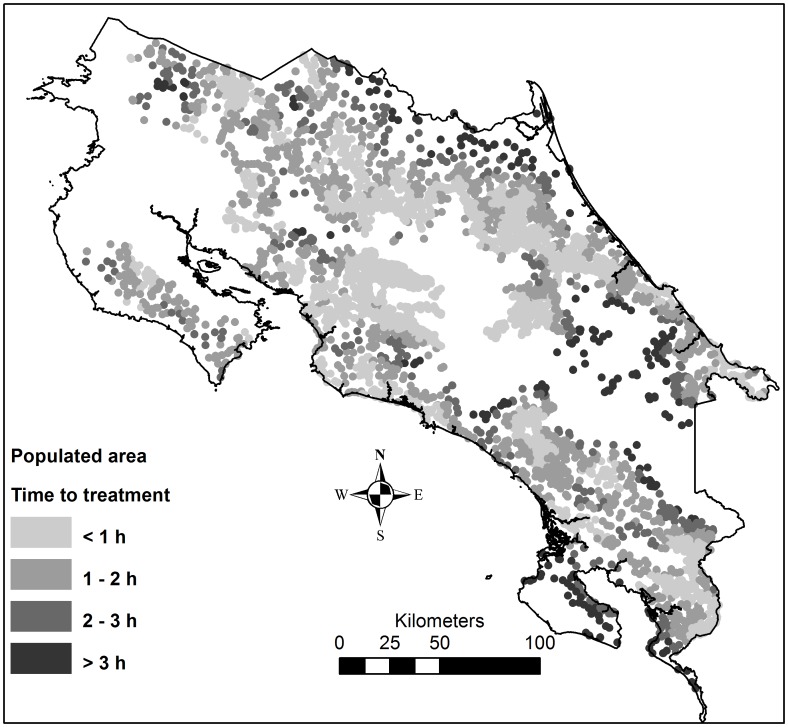
Time to hospital or clinic for populated areas at high risk of snakebites. Populated areas = areas within 2 km from a census tract centroid in a high risk of snakebites.

**Figure 7:**
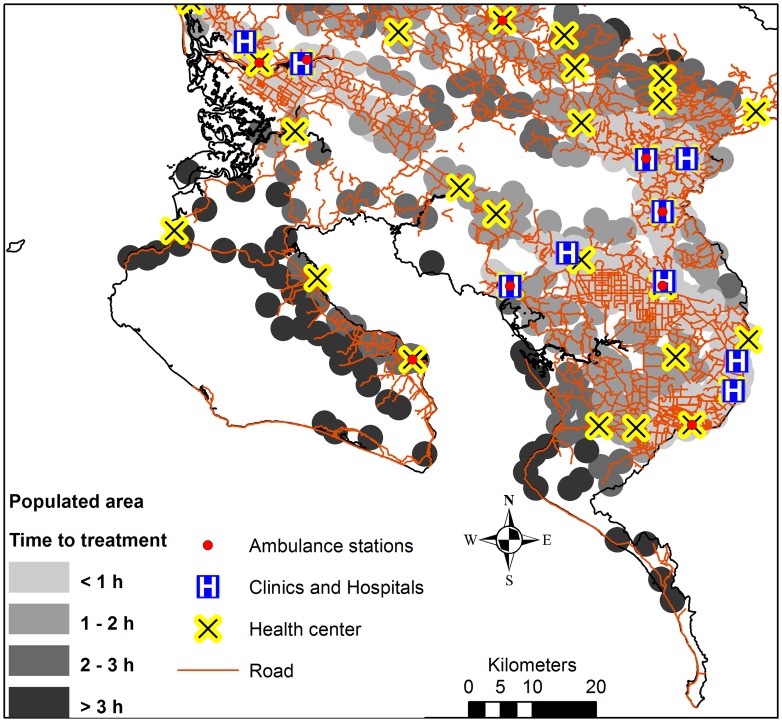
Close-up view of the Golfo Dulce region. Time to reach hospital or clinic for populated areas with a high snakebite risk, health care facilities and roads. Populated areas = areas within 2 km from a census tract centroid in a high risk of snakebites.

The time needed to go from an ambulance station to any cell, and the time to go from a hospital or clinic to any cell was calculated. These two time rasters were then summed to give a raster of the total time needed to reach healthcare facilities using ambulance, i.e. from ambulance station to snakebite victim and from there to healthcare (Figure 5). The mean time to reach healthcare (Figure 5) from populated areas in a high risk of snakebites (Figure 4) was extracted, and categorized into <2 h, 2–3 h and >3 h, in order to visualize the need of improved antivenom accessibility (Figure 6). Finally, the location of EBAIS and roads were added to the map of populations in need of improved antivenom accessibility, to develop small-scale maps of areas in need of improved accessibility to antivenom, which can be used to guide such improvements (Figure 7 and Supporting Information S1).

## Results

The non-spatial descriptive statistics of major parts of our dataset correspond very closely to what has been described in detail previously [14], and thus only a brief summary is presented in this article. A total of 9,333 cases were reported for the period 1990–2007, corresponding to an average incidence of 13.8 snakebites per 100,000 inhabitants per year. Seventy-two percent of the victims were male. Regarding age, 27% of victims were below 15 years of age, 32% between 15 and 30 years, 21% between 30 and 45 years, and the remaining 22% corresponded to people older than 45 years.

### District-level risk factors of snakebites

In multivariate analysis, lowland districts with much precipitation and few dry months generally had a higher snakebite incidence, as did districts with many rural inhabitants, and many inhabitants residing close to larger forests, although the last explanatory factor did not reach 95% significance (Table 1).

**Table 1:** District-level risk factors for snakebite incidence in multivariate Poisson regression.

Covariate	Unit	Incidence Ratio	95% Cr.I. Low	95% Cr.I. High
Elevation	+550 m	0.60	0.48	0.75
Dry months	+1.25 months	0.66	0.51	0.86
Precipitation	+827 mm/year	1.45	1.13	1.80
Population living within 500 m from >5 ha forest	+100%-units	1.53	0.88	2.59
Urban population	+100%-units	0.39	0.29	0.53

### Demonstration of spatial smoothing of small-area snakebite incidence data

The unsmoothed incidence observed in a one-year period had an acceptable ability to identify which districts would have a high snakebite incidence in the next five-year period (Table 2), but discriminatory ability was clearly improved by using smoothing. There were only minor differences between the estimates produced by the different smoothing methods A and B in discriminatory performance. When the training time was only one the incidence threshold exceedance probability of method A had a tendency to be better than the smoothed incidence produced by method B (p = 0.07). When the training time was five years, both methods had a borderline significantly better discriminatory ability than the unsmoothed incidence observed in five years (p = 0.02 for method A and p = 0.10 for method B).

**Table 2.Mean:** discriminatory abilities of the estimates produced by the two smoothing methods (A and B) for the two training time lengths (1 and 5 years).

Test statistic			95% Empirical C.I.	
Trainingtime (years)	Smoothingmethod	mean AUC	Low	High
1	None	0.83	0.79	0.86
5	None	0.93	0.91	0.95
1	A	0.95	0.93	0.96
5	A	0.96	0.95	0.97
1	B	0.93	0.91	0.95
5	B	0.96	0.94	0.97

A. The smoothed incidence exceedance probability estimate obtained from the Bayesian Poisson regression model with spatially correlated random effects and fixed effect explanatory variables, described in Methods section. B. The smoothed incidence estimate obtained from the built-in Bayesian spatial smoothing tool “Suavizador espacial de tasas” in SIGEpi [22].

### Identification of high-risk districts

There were marked geographical differences in the reported incidence, with high rates especially along the southern Pacific coast, and in parts of the Caribbean and northern lowlands (Figure 1). The districts with a high incidence corresponded to a large extent to the humid lowland areas identified as suitable habitat for *B. asper* (Figure 4); 91% of the rural population in high-incidence districts lived in this type of environment. However, only 51% of the rural population in humid lowland areas lived in districts with a high snakebite incidence. According to the population distribution of the 2000 census, 18% of the total population lived in districts with a high snakebite incidence and an additional 13% lived in rural, humid lowlands in districts not reporting an incidence above the threshold.

### Identification of high-risk areas in need of improved treatment accessibility

Hospitals, clinics, ambulance stations and roads were strongly aggregated in the great metropolitan area around the cities of San José, Heredia, Cartago and Alajuela, where a major part of the population lives. There is however also a network of roads and healthcare facilities in the more peripheral regions of the country (Figure 5). Of the population living in areas with a high risk of snakebites, 92.5% were estimated to have a minimum transportation time of less than 2 hour to hospitals or clinics, 5% were estimated to delay 2–3 hours to hospitals or clinics, and 2.5% more have transportation times higher than 3 hours. On the south Pacific coast around Golfo Dulce, around the Talamanca highlands in the southeast, and along the northern border, there are populations in high risk of snakebites and with long transportation times to antivenom treatment (Figure 6). Figure 7 provides a close-up view of one target region (Golfo Dulce), including all the information presented in the previous maps, as well as the location of primary health care facilities (EBAIS). This map demonstrates the type of map that can be prepared and used to identify vulnerable places where access to hospitals or clinics is delayed, thus setting the stage for the design of more effective ways to guarantee a more rapid access to antivenom treatment at the local level. Similar maps for other target regions are available as a supplement (Supporting Information S1).

## Discussion

### Key results

The spatial distribution of district-level snakebite incidence in Costa Rica largely followed the expected pattern, based on previous studies and on the distribution of the most important venomous snake in the country, *B. asper*. Incidence was higher in rural, humid lowlands, notably in the southern part of the country [14]. Geographical accessibility to antivenom treatment was generally good, however, in some areas there is a need of improved treatment accessibility. These are areas where hospitals and clinics are located relatively far from some of the areas with a likely high snakebite incidence.

### Limitations

The potential bias introduced by using data reported from the healthcare system to analyze snakebite incidence, due to the use of traditional medicine and dysfunctional reporting routines, are well known from other parts of the world [9], [37]. However, in contrast to the situation in many other developing countries in Latin America and elsewhere, traditional medicine is not widely used in Costa Rica [38] and the formal health care system is well developed and largely accessible [23]. The percentage of births attended by skilled personnel was 98.7% in 2007 [39], highlighting a highly developed and utilized formal healthcare system. A survey among snakebite victims receiving treatment at hospitals in 1996 found that only 2.9% of them had received any type of empirical treatment before reaching formal healthcare attention [19]. The literacy rate in Costa Rica is 96% [39], and educational campaigns of various sorts over several decades have raised awareness about the importance of seeking formal healthcare after snakebites among the Costa Rican public [19]. Based on these facts, we assume that the degree of utilization of traditional healthcare after snakebites is very low in Costa Rica and that, on this basis, there will not be much underestimation of the true snakebite incidence in the statistics available from the Ministry of Health. Nevertheless, our data may still suffer from under- or misreporting because of reporting errors, such as missing discharge reports.

Assuming equal distribution of snakebite risk within districts is a strong assumption. Even if districts are the smallest administrative unit, several spatial processes could still lead to large incidence variations within districts. One specific example of where problems are likely to arise due to within-district variation is in districts with high proportion of urban residents. Even though snakebite incidence might be high among the rural population of such districts, this might go unnoticed due to the large urban population among which there are few snakebites. By mapping census enumeration units with risk factors favoring snakebite occurrence, the impact of the above two limitations can be reduced as this risk-factor based approach is not dependent on the quality of the gathered incidence data or arbitrary district divisions.

The information about the location of the health care system must be regarded with caution; previous centrally available information about primary health care facilities has been found incorrect in an earlier study [23]. Furthermore, the method of locating primary health care facilities (EBAIS) by matching facility and community names is not infallible as there was not always a community with the same name as the EBAIS. However, this was mostly a problem in the urban areas, and thus of smaller importance for this study. The data about roads was generally old, and as there has likely been some improvement in road availability and quality since the data was gathered, our estimates of the time needed to reach treatment might be biased towards overestimation.

The estimated transportation times to reach hospital or clinic were much lower than those observed in previous studies of the time to hospital treatment of snakebites in Costa Rica. In a hospital-based study of all snakebites in 1996 [19], the time to reach hospital was recorded for approximately 70% of the patients. Of these, 61% reached hospital within 3 hours and 20% after more than 5 hours. However, the estimates cannot be fully compared with the transportation times observed in this study as they were recorded at hospitals, meaning that a major proportion of the patients could have received antivenom treatment at clinics and subsequently been transferred to a hospital, something that would delay the time to reach hospital substantially. Saborio et al. [40] found that among children admitted to the hospital in Limon on the Caribbean coast in 1985–1995, 50% received medical treatment within 3 hours, whereas the mean time was 6.8 hours, indicating very long transportation times for some snakebite victims in this area, parts of which are also estimated to have long times to treatment by our model. Another reason why it could be incorrect to compare the estimated times with the time observed in these studies is that they are at least 15 years old, and there have been improvements in ambulance and health care facility accessibility, telecommunications, and possibly road network since then. It should however be further emphasized that our model aim at estimating the ideal time to reach treatment, and that in reality there could be several unaccounted-for causes of longer times, such as problems in communicating with ambulance stations, temporarily impassable roads or unavailable ambulance services, etc. Even though there is a discrepancy between the estimated and observed times to treatment, we consider that the time-to-treatment estimation model provides important hints about the location of areas where the accessibility to antivenom treatment is more difficult. If the minimum time to treatment is estimated to be 2 hours in our analysis, there is an imperative for improved accessibility as the actual time to treatment will probably be longer.

It is however important to remember that healthcare accessibility cannot be reduced to a purely spatial concern. Logistical issues, such as effective communication with ambulance facilities, availability of ambulances, and problems with other forms of transportation in the communities need to be taken into account as well. Furthermore, geographical accessibility is just one dimension of health care access [41]; economic, social and cultural dimensions need to be also considered, something that is easily missed when doing analyses based on maps only. Research gathering empirical evidence on the actual, current time needed to reach treatment, and determinants of this time, would provide important information for the identification of vulnerable regions and for improving access to snakebite treatment in Costa Rica.

### Interpretation

The estimated minimum times to reach antivenom treatment were generally short, compared to the actual times to reach health facilities after snakebites reported in previous studies in Costa Rica [19], [40]. However, our analysis allowed the identification of some areas where accessibility to antivenom treatment needs to be improved. The specific strategies to be implemented to accomplish this demand a case by case analysis on a local basis, but a feasible alternative might be the distribution of antivenoms to some EBAIS, the strengthening of the training of health staff in antivenom use, and the organization of the work in such a way that antivenom is available at all times. There is a risk of over-interpreting the messages transmitted through these maps and forget how sensitive it is to data errors and assumptions of, for example, road speeds. Based on these limitations, we advise that the maps should be interpreted with care, and that the expert knowledge of actual conditions provided by health care officials at a local level is also taken into account when making decisions about allocation of treatment resources.

### Generalizability

The snakebite incidence data available in this study, country-wide, based on the smallest administrative unit and probably reliable, are not available in many of the countries where this type of study needs to be conducted, owing to the large underestimation of snakebite incidence and mortality by hospital statistics [37], [42], [43]. For data available as small area counts, Bayesian smoothing techniques have a well-known ability to improve interpretability [26], as further demonstrated in this study. Thus, using Bayesian smoothing, the interpretability of the gathered data can be increased so that more accurate estimations of area-level incidence can be made from sparse data. Leynaud and Reati [21] used a spatial Bayesian smoothing technique available in SIGEpi [22]. We compared this technique with a Bayesian smoothing technique that also allowed for variation in district risk factor composition, and enabled estimation of the probability that an incidence threshold value was exceeded. We found that there were benefits of employing such smoothing techniques to improve the interpretability of the raw incidence when the data material was sparse, whereas there was no significant difference when the data material was increased (in this case five years of observation time instead of one year).

Large-scale approaches to identifying areas in need of antivenom could also benefit from using GIS. Available household-based incidence surveys, hospital and mortality records etc., could be mapped and used to construct geostatistical models which, based on the spatial variation of snakebite burden and its relationship with other spatially varying factors, predict snakebite burden in areas for which there is no data available. By taking this spatial approach, the sparse data available could be better utilised than predicting snakebite burden in non-surveyed areas by extrapolating information to country or Global Burden of Disease Region, as was the method used in the most recent review of global snakebite burden [1]. There has been an attempt to use such methods to map snakebite in West Africa [10], and useful methods have been further developed in studies predicting burden of other tropical diseases, such as soil-transmitted helminth infection [44] and malaria [45]. However, in order for these approaches to be feasible, there is still a large need for more data on snakebite burden, especially in sub-Saharan Africa, where a recent systematical review found only a small number of studies [46]. Large-scale studies such as those recently conducted to estimate snakebite mortality and incidence in India [43] and Bangladesh [37], respectively, provide an important source of data for producing snakebite burden maps, especially if the geographical coordinates of the survey clusters are available.

Mapping the availability of treatment is another challenge to implementing our method on a large scale; antivenom availability in many areas of low income countries is known to be poor [3], but information on antivenom availability on facility or even country level is to our knowledge not easily available, but requires further data collection.

GIS not only offers the possibility to improve the interpretation of incidence data through spatial smoothing, but also to identify areas which, on the basis of environmental risk factors, could be expected to have a high snakebite incidence. This approach could be especially useful when incidence data are considered unreliable. We compared the map of areas with an environment considered as suitable habitat for *Bothrops asper*, with a map of specimen collection locations, and found that these had good congruence, except for the Nicoya Peninsula, where environmental degradation could have led to species disappearance [17]. The method of determining high-risk areas by environmental determinants requires prior knowledge about the habitat of the snake species and should ideally be complemented by such field studies of actual snake distribution. Another important application of GIS in the struggle for reducing the impact of snakebite envenoming is the ability to analyze geographical accessibility to treatment, an important factor for the outcome of the bite. Leynaud and Reati [21] used Euclidean distances to hospitals and roads to analyze access to treatment, a common and readily implemented choice. We used a more demanding method aiming at estimating the time required for a snakebite victim to reach healthcare that takes into account topography, land use, road type and location of ambulance services. This theoretically allows for a more detailed analysis of the time needed to reach treatment, especially in mountainous study areas.

Our study demonstrates that using GIS it is possible to facilitate rational decision-making on localization of treatment resources against snakebite by overlaying the risk of snakebite accidents, estimated using reported data and/or presence of risk factors, transport times to existing hospitals or clinics, and the location of possible additional facilities to which treatment resources could be allocated. GIS is a promising tool for devising cost-effective interventions aimed at reducing the public health impact of snakebite envenoming.

## Supporting Information

Supporting Information S1 **Close-up views of other areas at high risk of snakebite and low antivenom accessibility.**
(DOC)
