# Supplementary material for: Using Geographical Information Systems to Identify Populations in Need of Improved Accessibility to Antivenom Treatment for Snakebite Envenoming in Costa Rica
Source: PLoS Negl Trop Dis. 2013 Jan 31;7(1):e2009. doi: 10.1371/journal.pntd.0002009 (PMC3561131; doi:10.1371/journal.pntd.0002009)
Supplement: Supporting Information S1 — Close-up views of other areas at high risk of snakebite and low antivenom accessibility. (DOC) [file pntd.0002009.s001.doc]

# Supplement 1

#
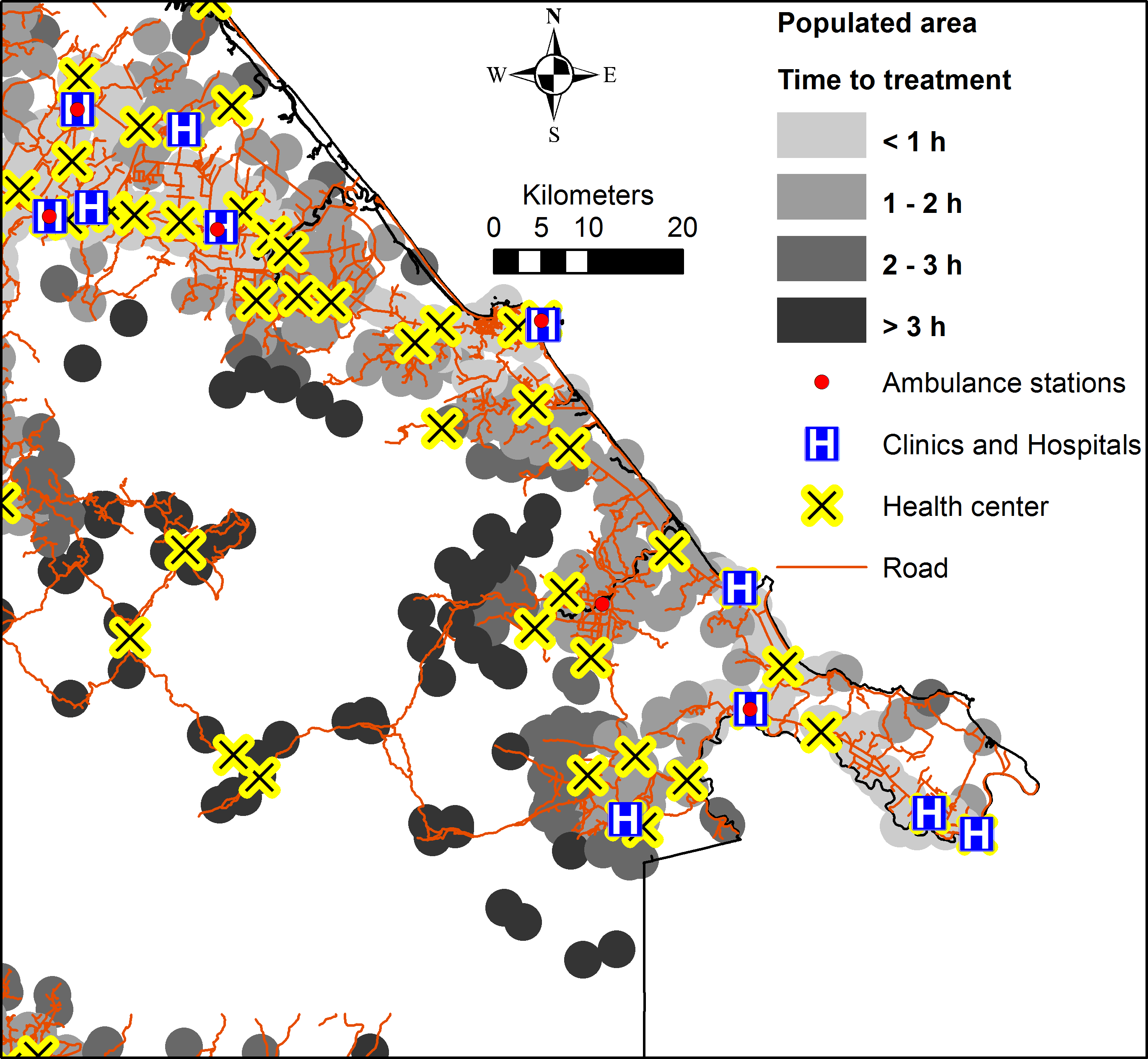


**Figure S1 Close-up view of the Talamanca region.** Time to reach hospital or clinic for populated areas with a high snakebite risk, health care facilities and roads. Populated areas = areas within 2 km from a census tract centroid in a high risk of snakebites.

#
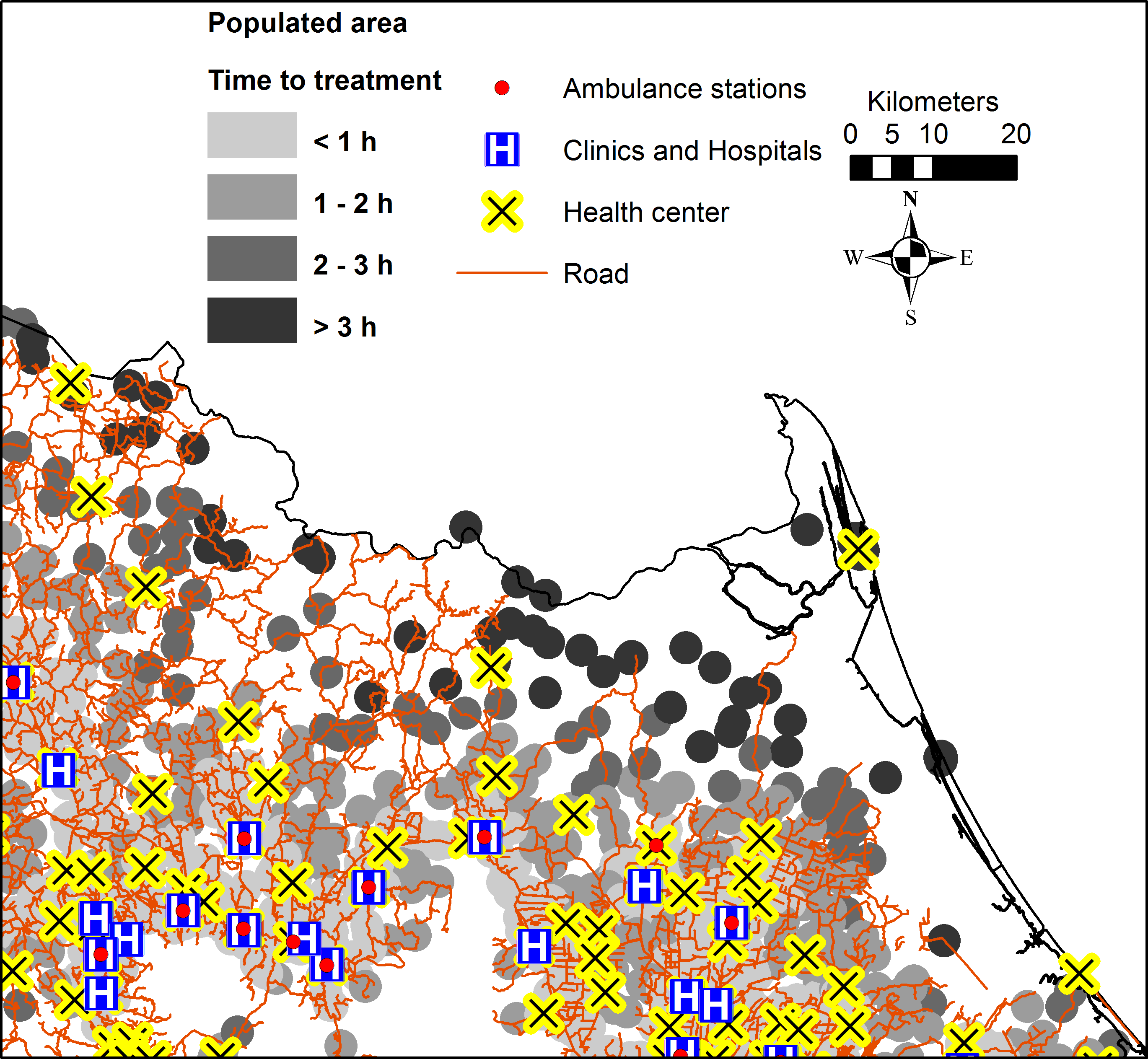


**Figure S2 Close-up view of the Northeast region**. Time to reach hospital or clinic for populated areas with a high snakebite risk, health care facilities and roads. Populated areas = areas within 2 km from a census tract centroid in a high risk of snakebites.

#
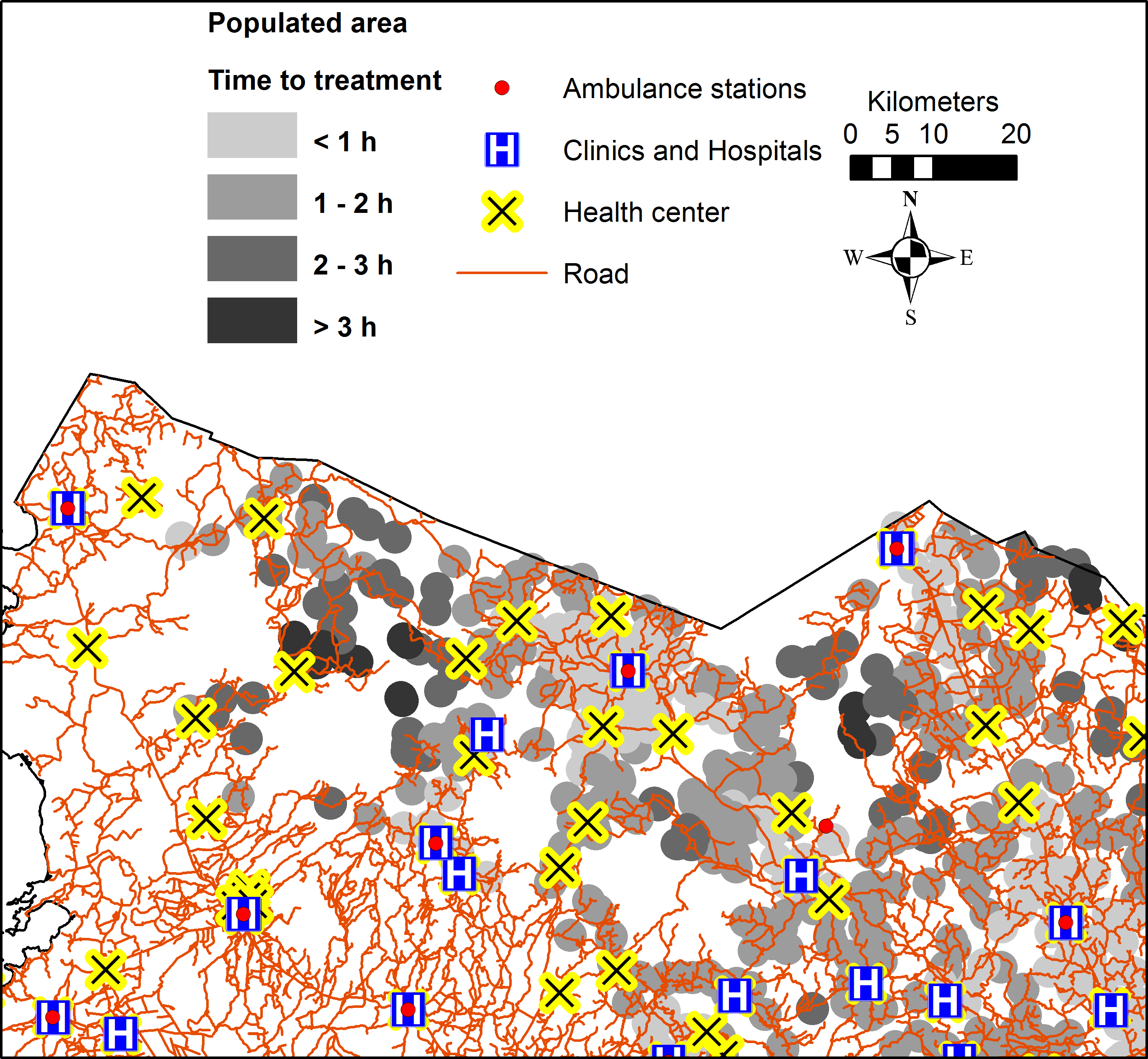


**Figure S3 Close-up view of the Northwest region.** Time to reach hospital or clinic for populated areas with a high snakebite risk, health care facilities and roads. Populated areas = areas within 2 km from a census tract centroid in a high risk of snakebites.
